# Two Eruptive Calcified Nodules in the Same Artery

**DOI:** 10.1002/ccr3.73648

**Published:** 2026-10-24

**Authors:** Ioannis Andreou, Manel Sabaté

**Affiliations:** ^1^ Hemodynamic and Interventional Cardiology Unit Sismanoglion Hospital Athens Greece; ^2^ University Hospital of Bellvitge Barcelona Spain; ^3^ IDIBELL Barcelona Spain; ^4^ CIBER‐CV Madrid Spain; ^5^ University of Barcelona Barcelona Spain

**Keywords:** acute coronary syndrome, calcified lesion, intravascular lithotripsy, optical coherence tomography, percutaneous coronary intervention

## Abstract

Simultaneous calcified nodule destabilization may occur in a multifocal manner, analogous to the phenomenon of multiple plaque ruptures, leading to an acute coronary syndrome. Optical coherence tomography can identify multiple eruptive calcified nodules in the same coronary artery and can influence treatment planning.

**Abbreviation**
CN: calcified nodule

A 64‐year‐old man, ex‐smoker with hypertension, dyslipidemia, and atrial fibrillation presented with chest pain due to inferior ST‐elevation myocardial infarction. Five years ago, he underwent percutaneous coronary intervention of the proximal right coronary artery with implantation of a 2.75 × 8 mm drug‐eluting stent. The patient was on optimal medical treatment and dabigatran. His left ventricular ejection fraction was 60%. His low‐density lipoprotein cholesterol levels on rosuvastatin 40 mg/day were 63 mg/dL.

In the current admission, right coronary artery angiography showed severely calcified diffuse disease with consecutive intermediate stenoses in the proximal and mid segments and a significant stenosis in the mid‐distal segment (Figure 1 and Video S1). Due to the extent of calcification, which hindered visualization of the previous stent, and the presence of diffuse disease with multiple stenoses, optical coherence tomography was performed. This revealed two eruptive calcified nodules (CNs): one in the proximal segment, just distal to the previously implanted stent, and a second in the mid‐distal segment (Figure 1 and Video S2). Coronary intravascular lithotripsy (Shockwave Medical, Santa Clara, CA) was used due to the presence of thick circumferential calcium, with sequential pulse treatments with 3.0 × 12 mm and 3.5 × 12 mm balloons applied from mid‐distal to proximal right coronary artery. Intravascular lithotripsy was chosen due to the presence of thick circumferential calcium along with deep medial calcification, with the aim of facilitating adequate lesion preparation and stent expansion. Given the diffuse calcified disease extending beyond the two prominent nodules, the entire calcified segment was treated rather than only the visually apparent nodules. After dilatation with non‐compliant balloons, two overlapping drug‐eluting stents were implanted (3.0 × 48 mm and 4.0 × 32 mm) (Video S3). The patient remains asymptomatic and event‐free at 4‐year follow‐up.

**FIGURE 1:**
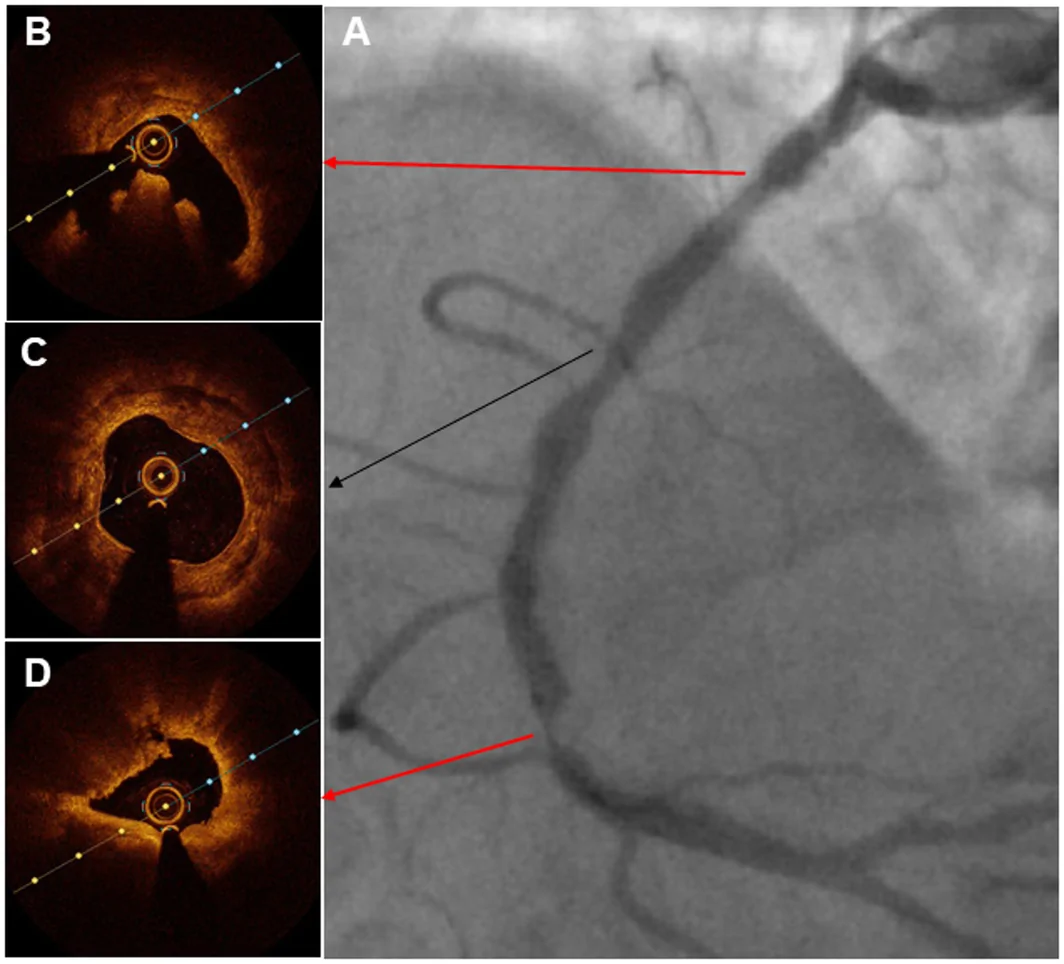
Coronary angiography and corresponding optical coherence tomography findings. Right coronary artery angiography (A) with optical coherence tomography cross‐sections demonstrating: the proximal eruptive calcified nodule (B), a concentric superficial calcified plaque (C), and the distal eruptive calcified nodule (D).

Eruptive CNs, the least frequent (∼5%) morphology causing thrombotic acute coronary syndrome, are characterized by a disrupted fibrous cap, often accompanied by an overlying fibrin/platelet thrombus, resulting in an irregular surface [1]. To the best of our knowledge, this is the first reported case of two eruptive CNs located within the same artery in a patient presenting with a myocardial infarction. This finding suggests that CN destabilization may occur concurrently, in a manner analogous to plaque rupture [2]. The simultaneous destabilization of calcified nodules in the same coronary artery may suggest a shared underlying pathophysiological process, potentially involving increased vascular inflammatory activity. However, this interpretation remains speculative and cannot be established from the findings of this single case. The distal calcified nodule was considered the presumed culprit lesion based on the angiographic findings and the associated thrombotic appearance. The proximal calcified nodule was regarded as a non‐culprit lesion, although its potential contribution to the acute coronary event cannot be completely excluded. CNs represent a challenging lesion subset with decreased procedural success and worse clinical outcomes, and the optimal treatment strategy has yet to be determined. Eruptive CNs are almost universally deformable, consistent with their composition of fragmented necrotic core calcification [1]. Although optimal disruption of calcium with intravascular lithotripsy is best achieved when the coronary calcification is circumferential, a recent optical coherence tomography analysis revealed that its application was safe and highly effective in treating CNs, leading to adequate stent expansion [3]. In a pooled analysis, the baseline minimal lumen area coincided with the location of the site of maximum CNs in only 51.7% of cases [3]. Additionally, in 57.7% of cases, after intervention the minimal stent area was located outside the maximum CN site [3]. Treatment approaches should target not only the CNs but also the entire calcified lesion [3]. Therefore, optical coherence tomography can identify multiple eruptive calcified nodules in the same coronary artery and can influence treatment planning.

## Author Contributions

**Ioannis Andreou:** conceptualization, writing – review and editing, supervision, writing – original draft. **Manel Sabaté:** conceptualization, supervision, writing – review and editing.

## Funding

The authors have nothing to report.

## Consent

Written informed consent for publication was obtained by the patient.

## Conflicts of Interest

The authors declare no conflicts of interest.

## Supporting information


**Video S1:** Initial angiography.

**Video S2:** Optical coherence tomography evaluation.

**Video S3:** Percutaneous coronary intervention and final angiography.

## Data Availability

Research data are not shared.
